# Use of BODIPY‐Cholesterol (TF‐Chol) for Visualizing Lysosomal Cholesterol Accumulation

**DOI:** 10.1111/tra.12414

**Published:** 2016-06-13

**Authors:** Maarit Hölttä‐Vuori, Erdinc Sezgin, Christian Eggeling, Elina Ikonen

**Affiliations:** ^1^ Faculty of Medicine, Department of AnatomyUniversity of Helsinki00014 HelsinkiFinland; ^2^ Minerva Foundation Institute for Medical Research00290 HelsinkiFinland; ^3^ MRC Human Immunology Unit, Weatherall Institute of Molecular MedicineUniversity of OxfordOX39DS OxfordUK

**Keywords:** BODIPY‐cholesterol, cholesterol accumulation, lipid imaging, Niemann–Pick type C disease, TF‐cholesterol

## Abstract

Dipyrromethene difluoride‐cholesterol (TopFluor‐Cholesterol, TF‐Chol) is a widely used cholesterol analogue due to its excellent fluorescence properties and considerable similarity with natural cholesterol in terms of membrane partitioning. However, the suitability of TF‐Chol for detecting lysosomal cholesterol deposition has recently been questioned. Here, we highlight the fact that the method of lipid delivery and the analysis of time‐point both affect the membrane distribution and labeling pattern of TF‐Chol, similarly as with radiolabeled cholesterol. Lysosomal sterol accumulation characteristic to a lysosomal storage disease is most readily detected when the probe is introduced via the physiological route, i.e. as a sterol fatty acid ester in low‐density lipoprotein particles. When administered to cells from solvent, lysosomal sterol sequestration becomes evident after an overnight equilibration between membranes.

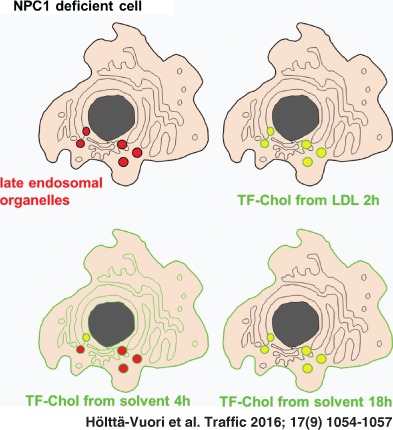

Recent advances in fluorescence microscopic techniques have sparked interest toward fluorescent lipid analogues that enable visualization of lipid distribution and trafficking in intact cells. These approaches are challenged by the fact that conjugation of fluorescent moieties tends to perturb lipid properties. This feature is highlighted in cholesterol, a small, hydrophobic and planar molecule. Cholesterol participates in the lateral ordering of the membrane, and even minor changes in its structure have been shown to alter this propensity 1, 2. In this regard, any fluorescent sterol analogue is unlikely to exhibit identical properties with natural cholesterol. Yet, desirable analogues would be ones that minimally perturb the behavior of the sterol and surrounding molecules and that can be sensitively detected.

One such probe now widely used is a dipyrromethene difluoride (BODIPY)‐labeled sterol analogue (TopFluor‐Cholesterol, TF‐Chol), originally designed by the group of Robert Bittman 3. TF‐Chol has a BODIPY moiety linked to carbon‐24 of the sterol side chain. Similarly to natural cholesterol, it partitions into ordered domains both in model and cell membranes 3, 4, 5. However, despite the proposed value of the probe, its suitability to trace cellular cholesterol deposition, in particular within lysosomes, has recently been questioned 5. At present, the most commonly used method of delivering TF‐Chol (and other sterol analogues) to cells is by dissolving in solvent and adding directly to the growth medium. With this approach, the sterol diffuses to cells from solvent, equilibrating slowly with intracellular membranes. This process is complicated by the fact that hydrophobic sterols such as TF‐Chol have the propensity to aggregate in an aqueous environment 4, 5.

A traditional test case for the cellular partitioning of cholesterol analogues has been Niemann–Pick type C (NPC), a lysosomal storage disorder that results in the accumulation of cholesterol in late endosomal compartments 6. Thus, visualization of the lysosomal cholesterol deposition of NPC cells has become one of the standard experiments to test how physiologically sterol analogues behave in cells. In 4 h upon administration from solvent, TF‐Chol can be visualized on the plasma membrane and diffusely in intracellular membranes (Figure 1A). At this time‐point, the probe does not yet show appreciable lysosomal sequestration in either control or NPC cells, as evidenced by colocalization analysis of TF‐Chol with lysosomal dextran 5 (Figure 1A, C). To visualize TF‐Chol accumulation in lysosomes with this labeling method, it is advisable to use incubation times of 18 h or longer 4 (Figure 1B, C). This is in line with studies on radiolabeled cholesterol: when delivered directly from the medium to NPC cells, the equilibration of [^3^H]‐cholesterol between the plasma membrane and lysosomal pools has an apparent half‐time of 35 h 7.

**Figure 1:**
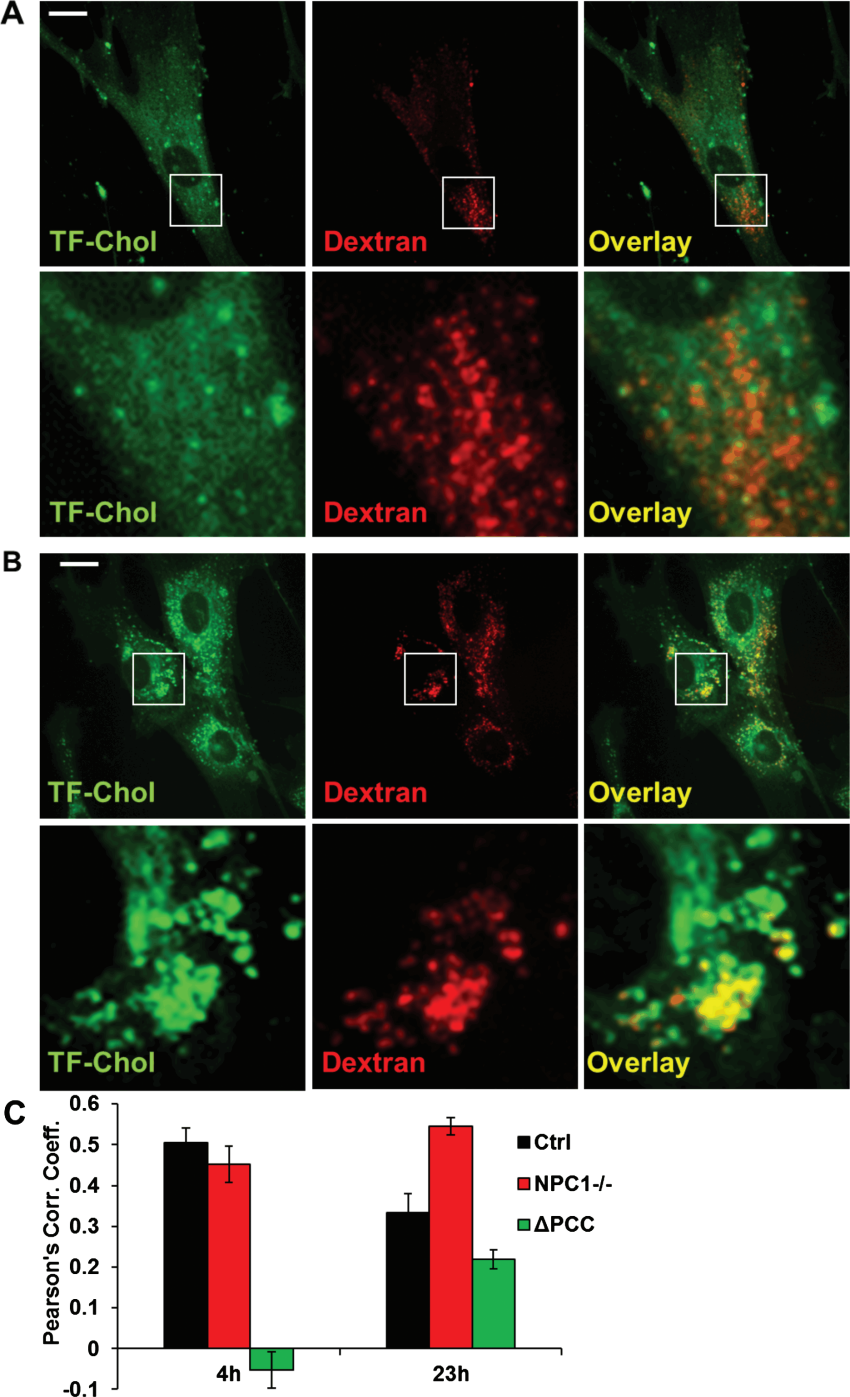
**Time‐dependent partitioning of TF‐Chol in the lysosomes of human primary fibroblasts**. NPC1‐deficient GM3123 fibroblasts (obtained from Coriell cell repository) were incubated with Rhodamine‐dextran overnight to label terminal endocytic compartments, followed by incubation with (A) 5 μg/mL (8.7 μm) TF‐Chol in normal growth medium containing 5% fetal calf serum for 4 h (as in Ref. 5), or (B) 0.5 μm TF‐Chol in medium containing 5% lipoprotein‐starved serum for 23 h (as in Ref. 6). Cells were imaged post‐fixation with a Nikon Eclipse Ti‐E Inverted Microscope (Nikon). Boxed areas are shown with higher magnification, scale bars 20 μm. C) Colocalization of TF‐Chol and dextran in control (ctrl) or NPC1‐deficient (NPC1 ^−/−^) cells was quantified from micrographs by using Pearson's correlation coefficient (PCC) (as in Ref. 5, values of 0 and 1 depict low and high colocalization, respectively). ΔPCC demonstrates the difference in the correlation coefficient in control versus NPC1‐deficient cells (values >0 depict an increase in colocalization). Error bars: SEM (N of cells: 5–9).

Major differences in the behavior of TF‐Chol and radiolabeled cholesterol in cells are increased efflux and decreased esterification of TF‐Chol compared to [^3^H]‐cholesterol 4. The BODIPY moiety of TF‐Chol has a slight disordering effect in disordered membrane domains, enhancing probe protrusion modes and likely contributing to sterol efflux at the expense of esterification. As TF‐Chol is readily effluxed from cells 4, prolonged incubation in the presence of efflux acceptors may significantly reduce the signal intensity. Therefore, it is preferable to use lipoprotein‐deprived serum during labeling from solvent carriers to reduce back‐exchange of the probe. Otherwise, one has to use a higher concentration of TF‐Chol, which increases its aggregation tendency 5.

TF‐Chol can also be delivered to cells from artificial or natural hydrophilic carriers. The probe can be readily complexed with beta‐cyclodextrin to perform short (e.g. 2 min) pulse labeling of the plasma membrane 4. This labeling method yields robust and uniform plasma membrane signal (at the level of light microscopy), compared to the slower and more asynchronous partitioning from solvent carriers. In terms of detecting lysosomal sterol deposition, the most physiological method to introduce TF‐Chol to cells is to incorporate it as a fatty acyl ester into low‐density lipoprotein (LDL) particles 8. In NPC cells, the hallmark is the inability of cells to transport LDL‐derived cholesterol out of late endosomal compartments, even though any cholesterol – also that added to the plasma membrane or endogenously synthesized – eventually becomes sequestered in the cholesterol‐laden endo/lysosomes 6. Deposition of LDL‐derived TF‐Chol in the lysosomes of NPC‐deficient cells is already evident upon 2 h of labeling, as this delivery route directly targets the compartment of the NPC defect 8. Upon lysosomal delivery, TF‐Chol ester is, similar to natural cholesterol, hydrolyzed by acid lipase and transported to cellular membranes in an NPC1‐dependent manner 8.

In summary, both the method of lipid delivery and the analysis of time‐point affect lipid partitioning in cellular membranes. In the case of TF‐Chol, lysosomal accumulation characteristic to NPC disease is most readily detected when the probe is introduced via the physiological route, i.e. as a sterol fatty acid ester in LDL particles. When administered to cells from solvent, lysosomal deposition becomes evident after an overnight equilibration between membranes.

## Supporting information

Editorial Process
