# Supplementary material for: Use of BODIPY‐Cholesterol (TF‐Chol) for Visualizing Lysosomal Cholesterol Accumulation
Source: Traffic. 2016 Jun 13;17(9):1054–7. doi: 10.1111/tra.12414 (PMC4988379; doi:10.1111/tra.12414)
Supplement: Supplementary file 1 — Editorial Process [file TRA-17-1054-s001.docx]

| Title: | **Use of BODIPY-cholesterol (TF-Chol) for visualizing lysosomal cholesterol accumulation** |
| --- | --- |
| Authors: | Maarit Hölttä-Vuori, Erdinc Sezgin, Christian Eggeling and Elina Ikonen |
| Article Type: | Traffic Interchange |
|  |  |
| Monitoring Editor | Sharon Tooze |
| Date Submitted | 3 May 2016 |
| Date for Decision 1 | 6 May 2016 |
| Date Resubmitted | 12 May 2016 |
| Accepted | 13 May 2016 |

**Decision and Reviews**

Dear Dr. Hölttä-Vuori

Thank you for submitting your manuscript “Use of BODIPY-cholesterol (TF-Chol) for visualizing lysosomal cholesterol accumulation” for consideration for publication in Traffic. I asked a colleague who is an expert in the field to review the paper and his/her verbatim comments are appended below. I agree with the referee that this is a very useful paper for the community. The referee has made a recommendation to clarify the purpose of the paper that I encourage you to include. In addition, I agree with the referee’s assessment that this paper is better suited as a Traffic Interchange than a Transit Authority. With these changes I would be pleased to accept this paper for publication in Traffic.

Sincerely,

Sharon A. Tooze, Ph.D.
Co-Editor

________________________________________________________

Referee's Comments to the Authors


Referee: 1

Comments to the Author
This short article resolves an apparent discrepancy in the literature in which the same cholesterol probe, BODIPY-cholesterol or TF-cholesterol, was used in two independent studies but gave different cellular labelling patterns. To their credit, the authors of those two studies have now co-authored this new manuscript and very clearly explain the differences in labelling that were reported. In addition, some practical aspects for using the cholesterol probe are provided.

This is a widely used probe and I believe that this article is entirely appropriate for publication. In my view it would be appropriate for a Traffic Interchange article. My only minor comment is that upon first reading the article I did not appreciate why the article was being written. In my view, it should be stated quite clearly in the second paragraph that despite the proposed value of the probe, there have been questions raised over its use as a marker of cellular cholesterol, and particularly, the labelling of lysosomes in NPC1-deficient cells (Ref 5). Although perhaps slightly less diplomatic, I think it is important to make clear why the article is being written and make sure the importance of the article is not missed.

_____________________________________________________________________________**Author Rebuttal**

Dear Dr. Hannan and Dr. Tooze,

We are here submitting a revised version of our article “Use of BODIPY-cholesterol (TF-Chol) for visualizing lysosomal cholesterol accumulation” by Maarit Hölttä-Vuori et al. (TRA-16-0520).

We were very pleased about the overall positive assessment of our manuscript and agree with your suggestion that this would be appropriate for a Traffic Interchange article. We have also modified the text according to Reviewer’s suggestion, to emphasize why the article was written (see Abstract and second paragraph, line 7). In addition, we have added an abstract, an abstract figure and a synopsis statement as suggested.

We thank the Reviewer and the Editors for the constructive remarks and hope that the revised manuscript is now acceptable for publication.

Sincerely yours,
Maarit Hölttä-Vuori and Elina Ikonen

_____________________________________________________________________________**Decision and Reviews Date**

_____________________________________________________________________________**Author Rebuttal Date**

_____________________________________________________________________________**Decision and Reviews Date**

_____________________________________________________________________________**Author Rebuttal Date**

_____________________________________________________________________________**Accepted and Sent to Press Date**
